# Repulsive Axon Guidance by Draxin Is Mediated by Protein Kinase B (Akt), Glycogen Synthase Kinase-3β (GSK-3β) and Microtubule-Associated Protein 1B

**DOI:** 10.1371/journal.pone.0119524

**Published:** 2015-03-16

**Authors:** Rajeshwari Meli, Petronela Weisová, Friedrich Propst

**Affiliations:** Department of Biochemistry and Cell Biology, Max F. Perutz Laboratories (MFPL), University of Vienna, Vienna Biocenter (VBC), 1030, Vienna, Austria; School of Biomedical Sciences, The University of Queensland, AUSTRALIA

## Abstract

Draxin is an important axon guidance cue necessary for the formation of forebrain commissures including the corpus callosum, but the molecular details of draxin signaling are unknown. To unravel how draxin signals are propagated we used murine cortical neurons and genetic and pharmacological approaches. We found that draxin-induced growth cone collapse critically depends on draxin receptors (deleted in colorectal cancer, DCC), inhibition of protein kinase B/Akt, activation of GSK-3β (glycogen synthase kinase-3β) and the presence of microtubule-associated protein MAP1B. This study, for the first time elucidates molecular events in draxin repulsion, links draxin and DCC to MAP1B and identifies a novel MAP1B-depenent GSK-3β pathway essential for chemo-repulsive axon guidance cue signaling.

## Introduction

During development of the nervous system growth cones at the tip of extending axons are guided to their targets with the help of guidance cues which can either attract or deflect axon growth [1]. Growth cone response to guidance cues is mediated by signal transduction pathways which have been characterized for several well-known guidance cues including netrin (an attractive or repulsive cue, depending on the molecular composition of the growth cone and the substratum [2]) and semaphorin 3A (repulsive [3,4]). Major signaling mechanisms include phosphatidylinositol 3-kinase (PI3K), phosphatase and tensin homolog on chromosome 10 (PTEN), glycogen synthase kinase-3β (GSK-3β) calcium, cAMP, cGMP and regulators of the growth cone cytoskeleton [1,5,6]. In contrast, little is known about the mechanisms mediating the response to draxin, a recently identified repulsive guidance cue [7]. Draxin is essential for guiding axons across the midline and draxin-deficient mice exhibit complete absence of all forebrain commissures including the corpus callosum [7]. In addition, draxin inhibits axon outgrowth and migration of embryonic murine cerebellar neurons [8] and directs axon projections in the optic tectum during chicken development [9]. Draxin interacts with high affinity with the netrin receptor deleted in colorectal cancer (DCC) [10], but how the draxin signal is propagated is not known.

A key target of guidance cue signaling is the growth cone cytoskeleton consisting of F-actin and microtubules [11]. Microtubules are regulated through a number of associated proteins including microtubule-associated protein MAP1B. MAP1B regulates the stability [12,13] and dynamics [14,15] of microtubules and has multiple roles in the formation of neuronal networks. It modulates axon branching and is necessary for directional growth cone migration [16]. It is needed for the neuronal responses to netrin-1 [17] and reelin [18], in the course of which it is phosphorylated by glycogen synthase kinase-3β (GSK-3β). It is also necessary for axon retraction in response to nitric oxide [19] and lysophosphatidic acid [20]. Mice deficient for MAP1B have defects in neuronal migration [21] and axon guidance [22]. The latter is evident from a failure to form the corpus callosum and the concomitant appearance of bundles of misguided cortical axons at the midline.

In the present study we set out to identify signal transduction pathways involved in draxin signaling. Given the similarity of phenotypes of draxin- and MAP1B-deficient mice we also investigated whether MAP1B mediates draxin-induced growth cone collapse and inhibition of axon extension. Using genetic and pharmacological approaches we were able to show that the PI3K/Akt/GSK-3β is necessary to transduce draxin signaling and that MAP1B is indeed involved.

## Materials and Methods

### Ethics Statement

The current study does not contain in vivo experiments using live animals. Newborn mice were sacrificed by decapitation in compliance with the Austrian law regulating the use of animals in biomedical research, Tierversuchsgesetz, BGBl. Nr. 501/1989 and BGBl. I Nr. 162/2005. Since no experiments on live animals were performed, approval of the experiments by the Institutional Animal Care and Use Committee was not required according to the above cited law (see also NC3Rs S1 Arrive Checklist). Animals for the production of newborn mice were housed at the in-house animal facility of the Max F. Perutz Laboratories which has been certified by the Austrian Federal Ministry of Science, Research and Economy (permit number BMWFW-66.006/0012-WF/II/3b/2014). Individual breeding pairs for the production of newborn mice were held in breeding cages according to the guidelines of the Federation of European Laboratory Animal Science Associations (FELASA).

### Cortical explants

Newborn MAP1B-/- mice [22] of either sex and wild-type controls were sacrificed by decapitation. Cerebral cortices of newborn pups were dissected in ice cold Hank’s balanced salt solution (Gibco), 7 mM HEPES pH 7.3 (Gibco) and 2 mM L-glutamine (Gibco), embedded in matrigel (BD Biosciences) and cultured in neurobasal medium (Invitrogen) supplemented with B27 (Invitrogen), 2 mM L-glutamine and penicillin/streptomycin (50 units/ml; Invitrogen) in the presence or absence of draxin (10 nM; R&D Systems) or semaphorin 3A (100 ng/ml R&D Systems). After 48 h images of live explants were taken using an Axio-observer microscope (Zeiss) and the length of the longest neurite of each explant was measured as described [10] using image J software.

### Primary neuronal cultures

Cortical neurons from newborn mice were isolated and cultured as described [23] with minor modifications. Cortical tissues were incubated with 0.25% trypsin, 0.02% EDTA (Sigma-Aldrich) at 37°C for 20 min followed by addition of Dulbecco’s modified Eagles medium containing Glutamax (DMEM; Gibco) with 10% fetal calf serum (FCS; Gibco) to inhibit trypsin activity. The cell suspension was triturated and cells were pelleted and re-suspended in DMEM with 5% FCS and 5% horse serum (Gibco). Dissociated neurons were plated for immunoblot analyses on poly-L-lysine (100 μg/ml, 1 h or overnight, 37°C) and laminin (20 μg/ml, 3 h, 37°C) coated plates at a density of 2x10^6^ cells per 35-mm culture dish or for growth cone collapse assays on poly-L lysine/laminin coated glass coverslips at a density of 5x10^4^ cells per 13-mm coverslip. After 2 h in culture, the medium was replaced with neurobasal medium supplemented with B27 and L-glutamine. Neuronal cultures were maintained at 37°C with 5% CO_2_ in a humidified chamber.

### Transfection of cortical neurons

1.5×10^6^ cells prepared as described above were re-suspended in 100 μl of nucleofector solution (Amaxa Mouse Neuron Nucleofector kit; LONZA). Cells were co-transfected with 0.75 μg of an expression vector encoding GFP (pmax-GFP; LONZA) to mark transfected cells and 2.25 μg of either pcDNA3 (empty vector control) or a construct encoding constitutively active Akt kinase (Akt-CA, full length mouse Akt1 with the 14-amino acid src myristoylation signal at its NH_2_ terminus [24]) or a construct encoding a dominant negative Akt mutant lacking kinase activity (Akt-DN, human Akt1 inactivated by a Lys179 to Ala mutation [25]). The Akt constructs were kindly provided by S. Pons (Biomedicine Institute, CSIC, Barcelona, Spain). Transfections were performed in an Amaxa transfection device (Amaxa Biosystems), using program G-013 according to the manufacturers specifications. Cells were plated and grown on poly-L-lysine/laminin coated coverslips as described above.

### Treatment of neurons

In general, treatments were carried out 60 h after plating except for experiments with transfected neurons where treatments were performed 26 h after transfection and plating to avoid the potential induction of apoptosis due to prolonged expression of Akt-DN [26]. Draxin was used at 100 nM for the times indicated, semaphorin 3A at 100 ng/ml for 30 min. In experiments using inhibitors cells were pretreated with inhibitor for 1 h prior to draxin treatment for 1 h. Inhibitors were the GSK-3 inhibitor SB216763 (Sigma-Aldrich) at 1 μM, the PI3K inhibitor wortmannin (Sigma-Aldrich) at 0.1 μM or the corresponding amount of DMSO as solvent control or anti-DCC function blocking antibodies (AF5; Calbiochem; [27]) at 1 μg/ml or 5 μg/ml. Mouse IgG1 (1 μg/ml or 5 μg/ml; Dako) was used as negative control.

### Growth cone collapse assay

Cortical neurons were grown on glass coverslips and treated as described above. Following treatment cells were fixed by addition to the medium of an equal volume of 8% paraformaldehyde, 22% sucrose and incubated for 20 min. Fixed cells were washed twice with PBS and permeabilized with 0.1% of Triton X-100 in PBS for 10 min. Cells were blocked with 2% BSA in PBS containing 0.01%Tween 20 for 1 h, followed by staining with Texas red-conjugated phalloidin (1:200; Molecular Probe) for 1 h for visualization of F-actin. Images were acquired on an Axio-observer microscope. Quantification was carried out as described [28]. Growth cone collapse was judged by the absence of F-actin rich lamellipodia at the tips of neurites and not more than two filopodia at the tip of the neurites. Only processes longer than 20 μm were evaluated [29]. In case of transfected cells only GFP positive cells were evaluated.

### Protein extracts and immunoblotting

Pelleted cells were lysed in RIPA buffer (Sigma-Aldrich) as described [23] in the presence of protease (1 cOmplete ULTRA Tablet, EDTA-free per 10 ml; Roche) and phosphatase (2 mM sodium orthovanadate (Sigma-Aldrich) and Phosphatase Inhibitor Cocktail (1:100; Sigma-Aldrich)) inhibitors. Protein lysates (25 μg/lane) were fractioned by denaturing sodium dodecyl sulfate polyacrylamide gel electrophoresis on 6% (for MAP1B analyses) or 10% (for all other analyses) gels and were transferred to nitrocellulose membrane (GE Healthcare Life Sciences). Membranes were blocked with 2% low fat milk (for MAP1B total, phospho-MAP1B and neurofilament H analyses) or 5% low fat milk (GSK-3β analyses) or 3% BSA (Akt analyses) in Tris buffered saline (TBS) containing 0.1% Tween 20. Primary antibodies: mouse monoclonal antibodies against phospho-MAP1B (SMI31; 1:1000; Covance) and neurofilament H (1:1000; Cell Signaling); rabbit polyclonal antibodies against total-MAP1B raised against peptides ATVVVEATEPEPSGC and ETVTEEHLRRAIGN [22], phospho Ser473 Akt (1:1000; Cell Signaling), total Akt (1:1000; Cell Signaling) and GAPDH (1:3000; Sigma-Aldrich); rabbit monoclonal antibodies against phospho Ser9-GSK-3β (1:1000; Cell Signaling) and total GSK-3β (1:1000; Cell Signaling). Anti-mouse and anti-rabbit horse radish peroxidase-conjugated secondary antibodies were used for detection (1:10,000; Jackson). Blots were developed using SuperSignal West Pico Chemiluminescent substrate (Thermo Scientific). Immunoblot images were acquired using X-ray films or the Fusion-FX7 Advance system (Peqlab). Quantification was performed by ImageJ version 1.44p software (National Institutes of Health, USA). Uncropped immunoblots are shown in S1 Fig.

### Statistical analysis

All values obtained in individual experiments are available in S1 Dataset. All statistical analysis was done with SPSS software (IBM) using one-way ANOVA and Tukey’s post hoc tests. The results are shown as mean values ± s.e.m.

## Results

Draxin-induced growth cone collapse and inhibition of neurite outgrowth are suppressed in MAP1B-deficient neurons

**Fig 1:**
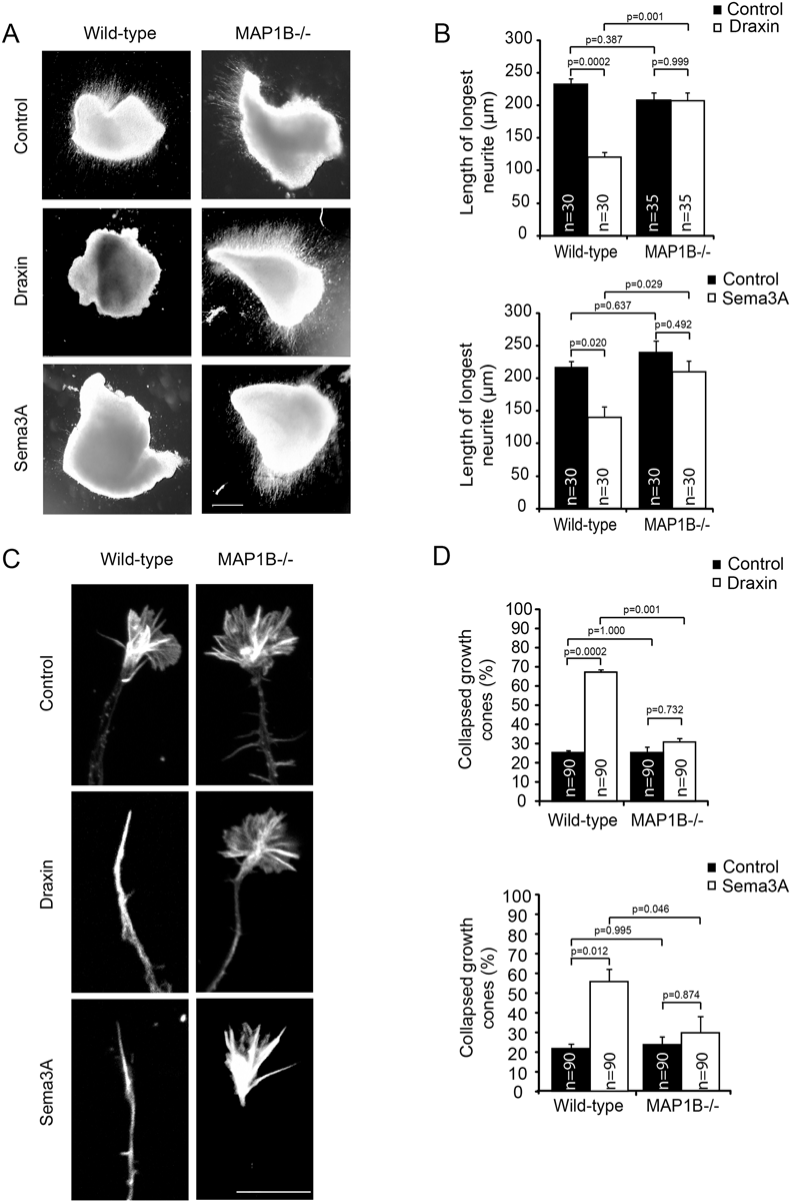
MAP1B is required for draxin- and semaphorin 3A-induced growth cone collapse and inhibition of neurite outgrowth. A. Cortical explants from newborn wild-type or MAP1B-/- mice, cultured for 48 h in the presence or absence (control) of 10 nM draxin or 100 ng/ml semaphorin 3A (Sema3A). Scale bar = 100 μm. B. Quantification of the longest neurites of 30 explants in 3 independent experiments. C. Growth cones of cortical neurons from newborn wild-type or MAP1B-/- mice cultured for 60 h, treated for 1 h with 100 nM draxin or 30 min with 100 ng/ml semaphorin 3A (Sema3A), fixed and stained for F-actin. Scale bar = 10 μm. D. Percentage of collapsed growth cones. For each experimental condition the growth cones of 30 neurons in each of 3 independent experiments were evaluated.

The similarity of corpus callosum and hippocampal commissure defects in draxin [7] and MAP1B mutant mice [22] prompted us to investigate the potential role of MAP1B in chemo-repulsive draxin signaling. Cortical explants from newborn wild-type and MAP1B-/- mice were grown in the presence or absence of draxin for 48 h prior to measurement of the length of extended neurites. Draxin inhibited neurite outgrowth in wild-type cortical explants whereas MAP1B-/- neurons were insensitive to draxin (Fig. 1A and B). To investigate the acute effect of draxin on growth cone migration we performed assays using isolated cortical neurons from newborn mice. Draxin induced growth cone collapse in over 60% of wild-type neurons (Fig. 1C and D). In MAP1B deficient neurons the draxin effect was not significant. Growth cone collapse in wild-type neurons was evident after 60 min of incubation, consistent with previous observations [10]. These results suggest that draxin-induced growth cone collapse and attenuation of neurite outgrowth are mediated by MAP1B. To test whether this role of MAP1B is specific for draxin or is shared by other repulsive guidance cues, we used an additional, well characterized repulsive guidance cue, semaphorin 3A, and obtained similar results (Fig. 1). Therefore, our data suggest that MAP1B is a general key component in mediating the effects of chemo-repulsive guidance cues.

### Draxin induces GSK-3β-dependent phosphorylation of MAP1B

**Fig 2:**
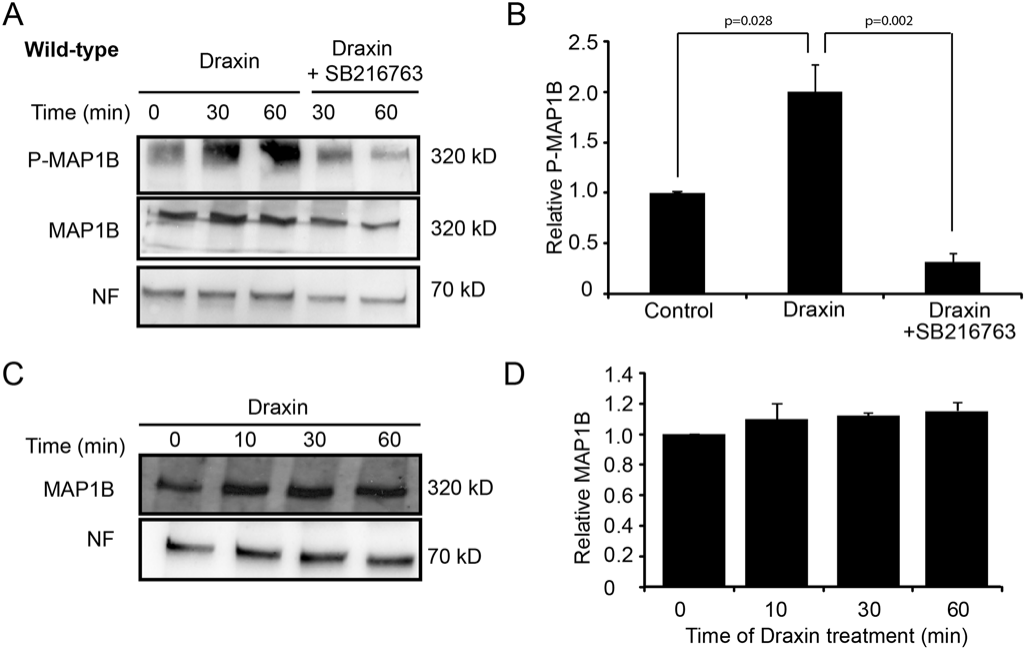
Draxin treatment increases the levels of GSK-3β-dependent phosphorylation of MAP1B on Ser1260. Immunoblot analyses of cortical neurons from newborn wild-type mice cultured for 60 h, treated with draxin in the absence or presence of the GSK-3β inhibitor SB216763 for the indicated times, lysed and probed using the indicated antibodies. A. Representative immunoblot for determination of MAP1B phosphorylation in response to draxin treatment. B. The relative level of phosphorylated MAP1B (P-MAP1B) was determined by normalizing the P-MAP1B signal to the signal for total MAP1B 30 min after draxin addition in 3 independent experiments. C. Representative immunoblot for determination of MAP1B protein levels in response to draxin treatment. D. The relative level of MAP1B was determined by normalizing the MAP1B signal to the signal for neurofilament H in 3 independent experiments. Draxin treatment did not lead to a significant change in MAP1B levels.

MAP1B is subject to posttranslational modification by GSK-3β which phosphorylates MAP1B at Ser1260 [30]. To test whether draxin induces phosphorylation of MAP1B at this site cortical neurons were treated with draxin for various times, lysed and analyzed by immunoblotting using a monoclonal antibody which recognizes the Ser1260 epitope exclusively in its phosphorylated form [30]. Draxin treatment increased the level of MAP1B phosphorylation (Fig. 2A). This increase in MAP1B phosphorylation was dependent on GSK-3 activity as it was inhibited by addition of SB216763, a GSK-3β inhibitor. Normalization of the signal for total MAP1B to the signal for neurofilament H revealed that draxin treatment did not change the levels of MAP1B protein (Fig. 2B). Together, these results show that draxin treatment of cortical neurons causes a rapid GSK-3-dependent increase in MAP1B phosphorylation with a time scale comparable to draxin induction of growth cone collapse.

### Draxin signaling involves activation of GSK-3β and inhibition of Akt

**Fig 3:**
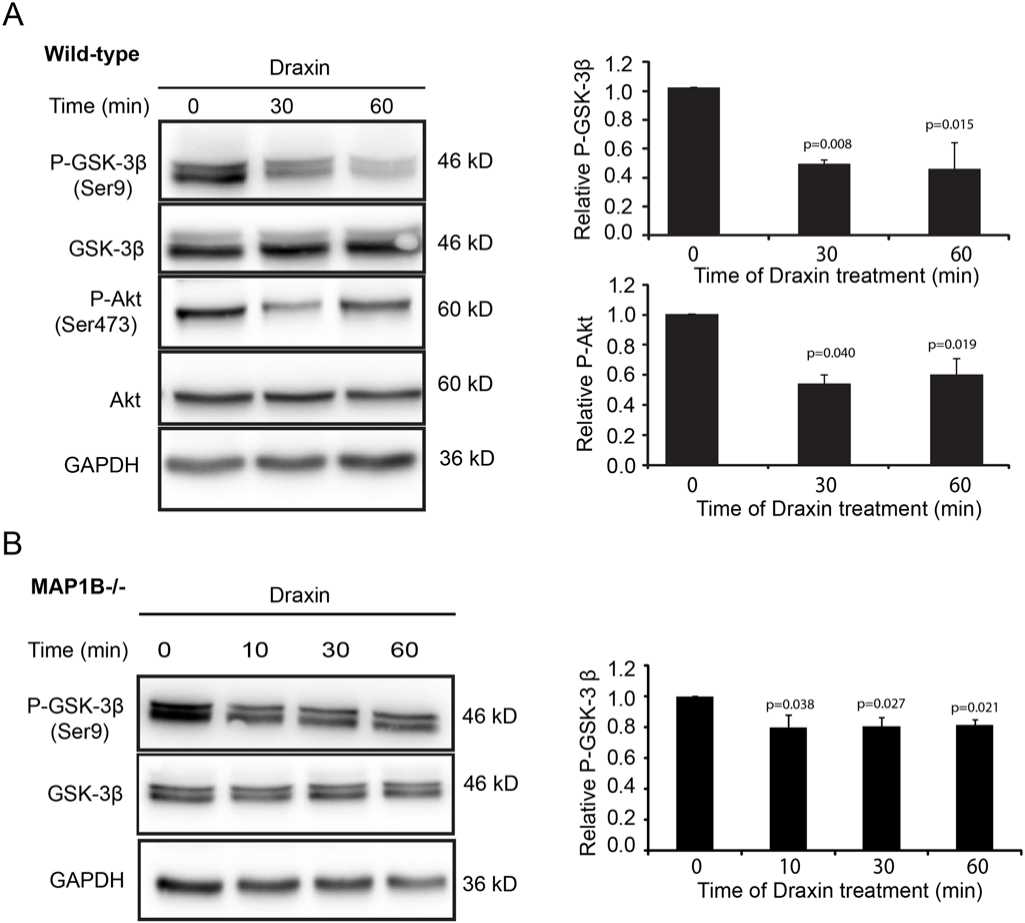
Draxin treatment of cortical neurons inhibits Akt activity and activates GSK-3β. Immunoblot analyses of cortical neurons from newborn wild-type (*A*) or MAP1B-/- (*B*) mice cultured for 60 h, treated with draxin for the indicated times, lysed and probed using the indicated antibodies. The GSK-3β doublets represent the GSK-3β1 and GSK-3β2 isoforms [33].The relative levels of GSK-3β phosphorylated at Ser9 (P-GSK-3β) and Akt phosphorylated on Ser473 (P-Akt) were determined by normalizing the signals for the phosphorylated proteins to the corresponding signals for the total proteins in 3 independent experiments.

We next analyzed whether the draxin-induced GSK-3-dependent increase in MAP1B phosphorylation correlates with an increase in GSK-3β activity. GSK-3β is negatively regulated by Ser9 phosphorylation [31,32]. We found that draxin treatment led to a fast and substantial reduction in Ser9 phosphorylation whereas total GSK-3β levels remained unchanged (Fig. 3A). The time scale of GSK-3β activation was compatible with the observed increase in MAP1B phosphorylation. A similar albeit reduced activation of GSK-3β by draxin was observed in MAP1B-/- neurons. This shows that draxin signaling upstream of GSK-3β leads to activation of GSK-3β even in the absence of MAP1B. However, in MAP1B-/- neurons activation of GSK-3β in response to draxin is attenuated (Fig. 3B).

In axon growth and guidance GSK-3β is a major downstream effector of PI3K and Akt with Akt negatively regulating GSK-3β by phosphorylating it on Ser9 [31,32]. Thus, our finding that GSK-3β is activated and dephosphorylated at Ser9 in response to draxin could be the result of inactivation of Akt. We tested this by analyzing the level of Akt phosphorylated on Ser473, a marker for active Akt [34]. Draxin treatment of cortical neurons resulted in a decrease in Akt phosphorylation at Ser473 (Fig. 3A), indicating that draxin signaling inhibited Akt, while the total levels of Akt remained unchanged. Together our data suggest that draxin treatment leads to inhibition of the PI3K/Akt pathway, resulting in activation of GSK-3β and an increase in the level of MAP1B phosphorylation.

### Draxin-induced growth cone collapse is dependent on activation of GSK-3β and inhibition of Akt

**Fig 4:**
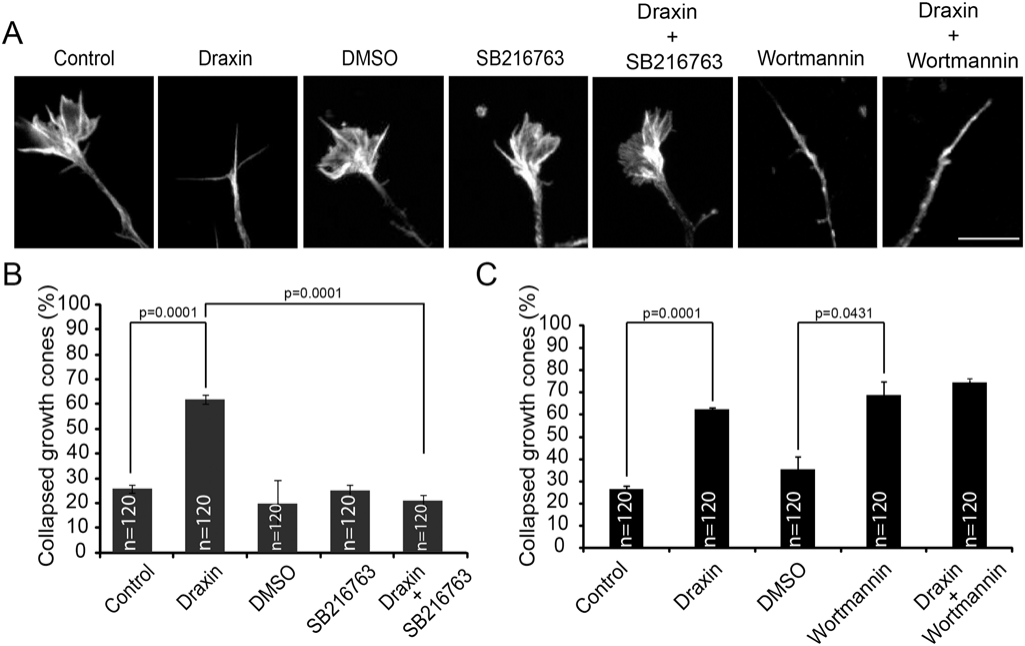
Draxin-induced growth cone collapse is dependent on GSK-3β activity and mimicked by inhibition of PI3K. A. Growth cones of cortical neurons from newborn wild-type mice cultured for 60 h, pre-treated with solvent (DMSO) or the GSK-3β inhibitor SB216763 or the PI3K inhibitor wortmannin for 1 h followed by addition of draxin for 1 h as indicated. Cells were fixed and stained for F-actin. Scale bar = 10 μm. B and C. Quantification of growth cone collapse. For each experimental condition the growth cones of 40 neurons in each of 3 independent experiments were evaluated.

To test whether GSK-3β activation is necessary for draxin-induced growth cone collapse we analyzed the effect of draxin in the presence of the specific GSK-3 inhibitor SB216763. Treatment with SB216763 had no effect on its own, but completely blocked the response of cortical neurons to draxin (Fig. 4A and B). These results demonstrate that GSK-3 activity is required for the biological activity of draxin.

Data presented in Fig. 2 showed that draxin treatment leads to a reduction in Akt activity. To explore the relevance of Akt inhibition for growth cone collapse we tested whether Akt inhibition by means other than draxin treatment can mimic the draxin effect. To this end we used wortmannin, an inhibitor of PI3K. PI3K generates phosphatidylinositol (3,4,5)-trisphosphate (PIP_3_) in the plasma membrane which leads to translocation and activation of Akt. Thus, inhibition of PI3K will result in inhibition of Akt. Wortmannin induced growth cone collapse in cortical neurons to about the same extent as draxin and simultaneous presence of draxin and wortmannin had no additional effect (Fig. 4A and C).

**Fig 5:**
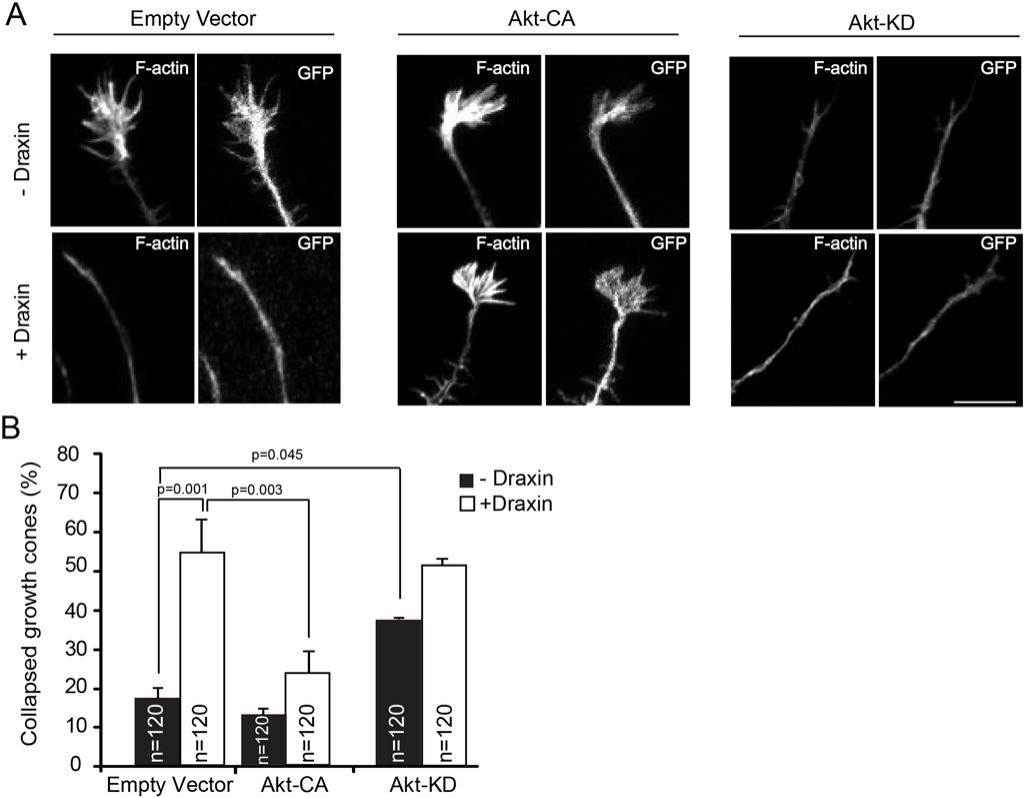
Draxin-induced growth cone collapse is prevented by constitutively active Akt. A. Growth cones of cortical neurons from newborn wild-type mice co-transfected with constructs encoding GFP and constitutively active (Akt-CA) or dominant negative (Akt-DN) Akt, cultured for 26 h and treated with or without draxin for 1 h as indicated. Cells were fixed and stained for F-actin. Scale bar = 10 μm. B. Quantification of growth cone collapse. For each experimental condition the growth cones of 40 neurons in each of 3 independent experiments were evaluated. C. Aliquots of transfected neurons analyzed for growth cone collapse in response to draxin were harvested in parallel for determination of Akt mutant expression by immunoblot using the indicated antibodies.

If inhibition of Akt is a necessary step in draxin signaling, cells ectopically expressing constitutively active Akt should be refractory to draxin treatment. We tested this prediction by co-transfecting cortical neurons with an expression construct encoding GFP (to identify transfected cells) and expression vectors encoding constitutively active (Akt-CA) or dominant negative Akt (Akt-DN). Cells co-transfected with the GFP construct and empty vector served as controls. Neurons were grown for 26 h, treated with draxin and analyzed in parallel for growth cone collapse and expression of the Akt mutant proteins. We found that in cells ectopically expressing constitutively active Akt draxin failed to induce growth cone collapse (Fig. 5). In contrast, cells expressing dominant negative Akt displayed increased growth cone collapse even in the absence of draxin. These results demonstrate that inhibition of the PI3K/Akt pathway is a critical step in draxin signaling.

### Draxin-induced growth cone collapse is mediated by DCC

**Fig 6:**
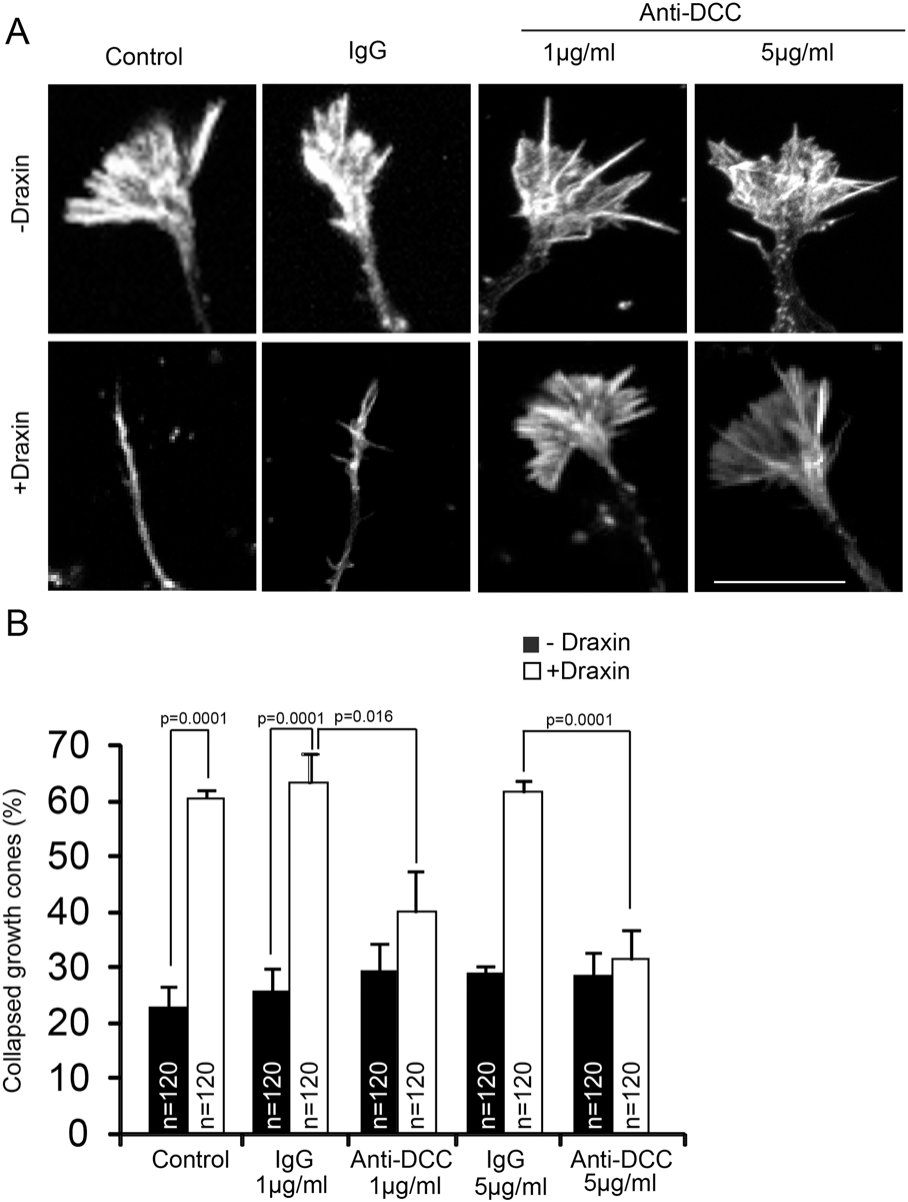
Draxin-induced growth cone collapse is mediated through DCC. A. Growth cones of cortical neurons from newborn mice cultured for 60 h, pretreated for 1 h with function blocking anti-DCC antibodies (anti-DCC) or unrelated IgGs at the indicated concentrations followed by addition of draxin for 1 h as indicated, fixation and staining for F-actin. Scale bar = 10 μm. B. Quantification of growth cone collapse. For each experimental condition the growth cones of 40 neurons in each of 3 independent experiments were evaluated.

Draxin has been shown to exert its effects on axon growth through the netrin receptor DCC [10]. To test whether draxin-induced growth cone collapse of cortical neurons is mediated through DCC we pretreated cortical neurons with a function blocking anti-DCC antibody [27] or control antibodies prior to exposure to draxin. Pretreatment with low concentrations of anti-DCC antibody blocked the induction of growth cone collapse by draxin, while the control antibody had no effect (Fig. 6).

## Discussion

**Fig 7:**
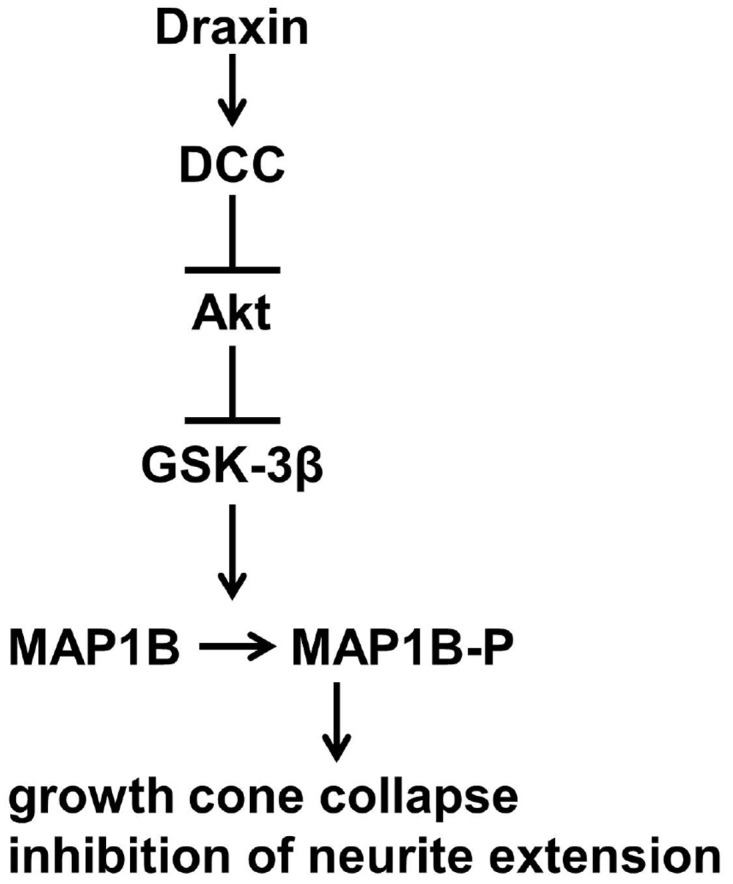
A model for repulsive draxin signaling. Draxin interaction with DCC triggers inactivation of the Akt pathway. This relieves GSK-3β from Akt-mediated inhibition leading to an increase in phosphorylation of MAP1B and reconfiguration of the cytoskeleton to promote growth cone collapse and inhibition of neurite extension.

Draxin has obtained significant interest as a novel chemo-repulsive guidance cue involved in development of the central nervous system, in particular the formation of forebrain commissures including the corpus callosum and the hippocampal commissure [7–10]. However, molecular mechanisms mediating its effects on growth cones and growing neurites have not been reported so far. As summarized in Fig. 7, our results demonstrate that draxin causes growth cone collapse through interaction with DCC, inhibition of the PI3K/Akt signaling pathway and activation of GSK-3β. GSK-3β enhances the level of MAP1B phosphorylation on Ser1260, a GSK-3β target site [30] and both, GSK-3 activity and MAP1B are necessary to convey the draxin signal. Thus, our study functionally links draxin, DCC and MAP1B. This might explain why ablation of any of the three genes leads to developmental defects in the formation of the corpus callosum and the hippocampal commissure [7,22,35]. The developmental defects in DCC and draxin knockout mice are more severe than those of MAP1B-deficient mice, and include defects in additional forebrain commissures and commissural axons in the spinal cord [7,35]. One potential explanation for these differences could be that both, draxin and DCC are acting upstream of MAP1B and thus might affect additional signaling pathways which are critical for commissure formation. These pathways might overcome MAP1B deficiency during development of some but not all commissures. For example, draxin has been shown to interact with UNC-5 [10], a co-receptor of DCC and modulator of DCC signaling [1]. It is conceivable that differences in UNC-5 expression during the development of individual commissures contribute to the different effects on formation of these commissures in draxin and DCC deficiency versus MAP1B deficiency.

The role of the PI3K/Akt pathway in neuronal morphogenesis and axon guidance signaling is well established [5]. For example, activation of PI3K/Akt signaling has been implicated in neuronal polarization. Likewise, neurotrophic factors including nerve growth factor (NGF) exert their effects through stimulation of the pathway [36] which is mediated through neurotrophin receptors tropomyosin related kinases (Trks) and p75NTR [37]. Conversely, growth cone collapse and inhibition of neurite growth by repulsive guidance cues such as semaphorins 3A, 3F and 4D and the growth inhibitory myelin component myelin-associated glycoprotein 1 have been associated with downregulation of the PI3K/Akt signaling pathway [38–41]. The crucial output of this pathway is the activity of Akt. Akt activity is directly linked to the level of PIP_3_ in the plasma membrane. PIP_3_ levels in turn reflect the relative activities of PI3K which synthesizes PIP_3_ and PTEN which hydrolyses it [6,42]. We show here that downregulating the PI3K/Akt pathway by direct inhibition of PI3K mimics draxin-induced growth cone collapse, extending previous observations with dorsal root ganglia explants [43] and neuroblastoma cells [44]. These findings emphasize the importance of downregulation of the PI3K/Akt pathway for growth cone collapse and neurite retraction, but they do not show that draxin inhibits PI3K. An alternative possibility would be that draxin stimulates PTEN. Indeed, this alternative has been implicated in induction of growth cone collapse by semaphorins 3A and 4D and the growth inhibitory myelin component myelin-associated glycoprotein 1 [39–41].

Our results raise the question as to how draxin might modulate PI3K/Akt signaling. One possibility is that draxin acts by binding to DCC. For one, we confirm a previous report [10] that draxin exerts its effects through DCC. Secondly, DCC has been linked to PI3K/Akt signaling in *Xenopus laevis* spinal neurons [45] and in *Caenorhabditis elegans* motor neurons [46]. However, in both of these paradigms netrin-1 binding to DCC lead to activation of the PI3K/Akt pathway, while draxin reduces Akt activity (Fig. 3) and the PI3K inhibitor wortmannin mimics its effects (Fig. 4). Moreover, the molecular details of the link between DCC and the PI3K/Akt pathway have yet to be elucidated.

MAP1B is essential for axon guidance by cues as diverse as netrin-1, reelin, lysophosphatidic acid and nitric oxide [17–20]. We show here that two additional chemo-repulsive cues, draxin and semaphorin 3A, depend on MAP1B for their action. In our model (Fig. 7) we depicted MAP1B in its canonical function as a microtubule regulator downstream of GSK-3β. However, we cannot exclude that MAP1B also impacts on draxin signaling upstream of GSK-3β, since we observed a decrease in GSK-3β activation in response to draxin in MAP1B-deficient as compared to wild-type neurons (Fig. 3A and B). Further studies will be needed to explore this interesting possibility.

Remarkably, both attractive (netrin-1) and repulsive (draxin) guidance cues trigger phosphorylation of MAP1B on Ser1260. This modification by itself does not change MAP1B interaction with microtubules [47], but correlates with changes in microtubule stability [47–49]. Together, these results suggest that MAP1B phosphorylation on Ser1260, although a critical event in growth cone response, is not involved in determining the outcome (growth cone collapse and axon retraction versus axon extension). Instead, it appears that MAP1B is a key component of a general mechanism which modulates neurite extension and growth cone collapse in response to attractive as well as repulsive guidance cue signals.

The essential role of GSK-3β in repulsive axon guidance has previously been characterized for semaphorin 3A [50]. Crucial GSK-3β targets in this respect are CRMP-2 [51–54] and CLASP [55], two proteins that regulate polymerization and stability of microtubules. We show here that growth cone response to semaphorin 3A and to draxin is also critically dependent on MAP1B. Thus, our results identify a third essential GSK-3β-dependent pathway that impacts on microtubules. Together, these findings demonstrate that GSK-3β regulates microtubules by a multipronged mechanism involving the phosphorylation of at least three microtubule regulators, further emphasizing that the precise regulation of microtubule dynamics is a key determinant in growth cone guidance.

## Supporting Information

S1 ARRIVE Checklist This table summarizes the compliance with the NC3Rs Arrive Guidelines. (PDF)

S1 Fig Uncropped immunoblots. Uncropped immunoblots for Fig. 2A and C, Fig. 3A and B and Fig. 5C. Lanes that are present on the blots but were not used in the figures are crossed out by thin lines. Blots display very low background and the appropriate protein bands are easily identifiable. In addition, the correct sizes of the protein bands were verified manually by comparing the bands to protein size markers visible in white light. The neurofilament (NF) blot of Fig. 2C was used as control for loading and transfer only.
(TIF)

S1 Dataset This file contains the individual-level data that were obtained in individual experiments (quantification of neurite outgrowth from explants, growth cone collapse and immunoblot quantifications) and are represented as summary statistics in Figs. 1, 2, 3, 4, 5 and 6. (XLSX)
