# Supplementary figures and images for: Repulsive Axon Guidance by Draxin Is Mediated by Protein Kinase B (Akt), Glycogen Synthase Kinase-3β (GSK-3β) and Microtubule-Associated Protein 1B
Source: PLoS One. 2015 Mar 16;10(3):e0119524. doi: 10.1371/journal.pone.0119524 (PMC4361590; doi:10.1371/journal.pone.0119524)

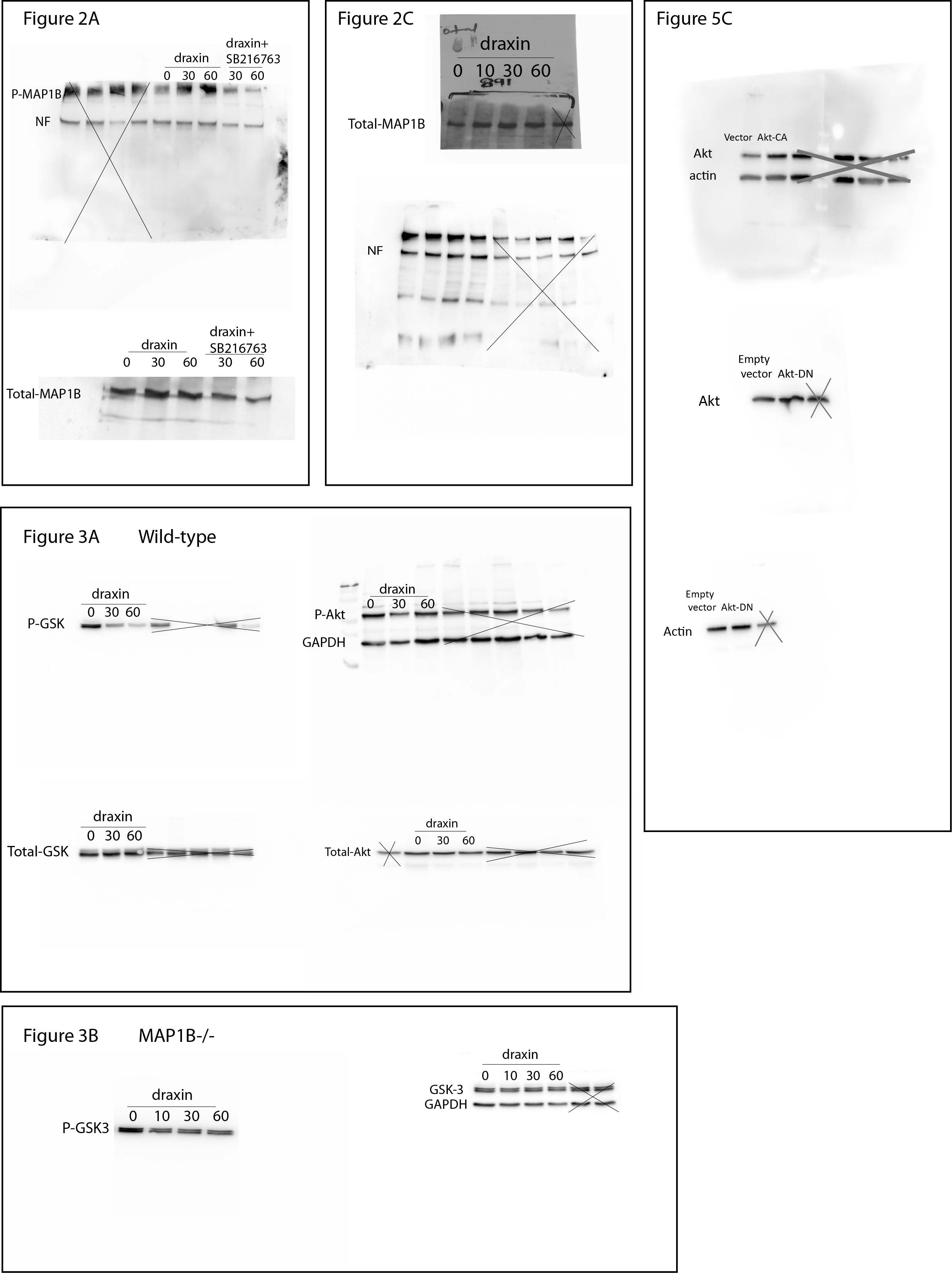

Supplement: S1 Fig — Uncropped immunoblots for Fig. 2A and C, Fig. 3A and B and Fig. 5C. Lanes that are present on the blots but were not used in the figures are crossed out by thin lines. Blots display very low background and the appropriate protein bands are easily identifiable. In addition, the correct sizes of the protein bands were verified manually by comparing the bands to protein size markers visible in white light. The neurofilament (NF) blot of Fig. 2C was used as control for loading and transfer only. (TIF) [file pone.0119524.s002.tif]
